# Prevalence of neglected tropical diseases in rural productive villages of the São Francisco River Integration Project in Ceará: cross-sectional study, 2020

**DOI:** 10.1590/S2237-96222025v34e20240393.en

**Published:** 2025-05-12

**Authors:** José Damião da Silva, Marta Cristhiany Cunha Pinheiro, Alberto Novaes Ramos, Bárbara Morgana da Silva, Anderson Fuentes Ferreira, Thainá Isabel Bessa de Andrade, Julieth Mesquita Lacerda, Letícia Pereira Araújo, Alanna Carla da Costa Belmino, Maria de Fátima Oliveira, Fernando Schemelzer de Moraes Bezerra

**Affiliations:** 1 Universidade Federal do Ceará, Faculdade de Medicina, Programa de Pós-Graduação em Saúde Pública, Fortaleza, CE, Brazil; 2 Universidade Federal do Ceará, Departamento de Análise Clínica e Toxicológica, Laboratório de Pesquisa em Parasitologia e Biologia de Moluscos, Fortaleza, CE, Brazil; 3 Universidade Federal do Ceará, Faculdade de Medicina, Departamento de Saúde Comunitária, Fortaleza, CE, Brazil; 4 Universidade Federal do Ceará, Departamento de Análises Clínicas e Toxicológicas, Laboratório de Pesquisa em Doença de Chagas, Fortaleza, CE, Brazil; 5 Universidade Federal do Ceará, Faculdade de Medicina, Programa de Pós-Graduação em Patologia, Fortaleza, CE, Brazil; 6 Universidade Federal do Ceará, Faculdade de Medicina, Programa de Pós-Graduação em Ciências Médicas, Fortaleza, CE, Brazil

**Keywords:** Neglected Diseases, Public Health Surveillance, Primary Health Care, Epidemiological Surveys, Cross-Sectional Studies, Enfermedades Desatendidas, Vigilancia en Salud Pública, Atención Primaria de Salud, Encuestas Epidemiológicas, Estudios Transversales

## Abstract

**Objective:**

To estimate the prevalence of Chagas disease, Hansen disease and schistosomiasis among residents of rural productive villages of the São Francisco River Integration Project in Ceará and to verify the presence, species and positivity of triatomines for *Trypanosoma cruzi*.

**Methods:**

This is a descriptive cross-sectional study conducted in the productive villages located in Jati, Brejo Santo and Mauriti, based on a clinical-epidemiological, serological and parasitological survey for schistosomiasis, Chagas disease and Hansen disease; and a triatomine survey. The descriptive analysis was composed by calculating absolute and relative frequencies with 95% confidence intervals.

**Results:**

The prevalence of schistosomiasis was 0.97% (2/206) by the Kato-Katz method and 11.54% (27/234) by the Immunochromatographic POC-CCA Test method. For Chagas disease, the prevalence was 0.27% (1/368). The suspected cases of Hansen disease through dermato-neurological examination comprised 2.67% (8/300) of the subjects, none of which were subsequently confirmed. Among the 245 household units investigated, triatomines were identified in 4 them (1.63%) (6 *Triatoma pseudomaculata* and 1 *Panstrongylus megistus*), but none with the presence of *Trypanosoma cruzi*.

**Conclusions:**

Chagas disease and schistosomiasis persist as endemic in these study areas. Even without the identification of triatomines infected by *T. cruzi* and of Hansen disease cases, the context of the region reinforces the need for continuous surveillance. It is essential to implement integrated public health actions to combat different neglected tropical diseases in new territories of human occupation. Contexts of endemicity and vulnerability make it essential to strengthen this topic on municipal and state public agendas.

## Resumen

**Objetivo:**

Estimar la prevalencia de la enfermedad de Chagas, la lepra y la esquistosomiasis entre los residentes de las aldeas rurales productivas del Proyecto de Integración del Río São Francisco en Ceará y verificar la presencia, las especies y la positividad de los triatominos para *Trypanosoma cruzi*.

**Métodos:**

Se trata de un estudio transversal descriptivo en las aldeas productivas ubicadas en Jati, Brejo Santo y Mauriti a partir de una encuesta clínica-epidemiológica, serológica y parasitológica para esquistosomiasis, enfermedad de Chagas y lepra; y una encuesta de triatominos. El análisis descriptivo se compuso mediante el cálculo de frecuencias absolutas y relativas con intervalos de confianza del 95%.

**Resultados:**

La prevalencia de esquistosomiasis fue del 0,97% (2/206) por el método Kato-Katz y del 11,54% (27/234) por el método inmunocromatográfico POC-CCA. Para la enfermedad de Chagas, fue del 0,27% (1/368). Se verificó un 2,67% (8/300) de casos sospechosos de lepra por examen dermatoneurológico, ninguno confirmado posteriormente. Entre las 245 unidades domiciliarias investigadas, en 4 (1,63%) se identificaron triatominos (6 *Triatoma pseudomaculata* y 1 *Panstrongylus megistus*), pero ninguno con presencia de T. cruzi.

**Conclusione:**

s: La enfermedad de Chagas y la esquistosomiasis persisten como endémicas en estas áreas de estudio. Incluso sin la identificación de triatominos infectados con *T. cruzi* y de casos de lepra, el contexto de la región refuerza la necesidad de una vigilancia continua. Es fundamental implementar acciones integradas de salud pública para combatir diferentes enfermedades tropicales desatendidas en nuevos territorios de ocupación humana. Contextos de endemicidad y vulnerabilidad hacen imprescindible fortalecer el tema en las agendas públicas municipales y de los estados.

## Introduction

The process of implementing large water projects invariably entails significant environmental, economic and sociocultural changes, which involve the most dissonant aspects of regional dynamics. This process interferes in the daily lives of the affected populations, involving deterritorialization and modification of their livelihoods, especially with regard to health conditions and quality of life (1,2).

In large water projects, it is essential to understand that damming water causes sudden changes in the ecosystem. This facilitates the emergence and spread of diseases, which may not, to date, be endemic in these regions (2,3). 

In this scenario, the São Francisco River Integration Project emerged, a federal government undertaking designed to guarantee water security by 2025 for 12 million inhabitants of small, medium and large cities in the semi-arid region of Northeast Brazil. To achieve this goal, two canals were built: North Axis, taking water to the hinterlands of Pernambuco, Ceará, Paraíba and Rio Grande do Norte, and East Axis, taking water to part of the hinterlands and agreste region of Pernambuco and Paraíba (2.4). 

The project raises major concerns in the context of public health, especially because it changes the social and economic conditions of the lives of people affected by the works, which may lead to an increase in the occurrence of diseases associated with poverty, such as neglected tropical diseases (2,5,6). These comprise a group of 23 diseases caused by viruses, bacteria, parasites or fungi, with an endemic pattern of occurrence associated with poverty (5,7).

This group of diseases continues to have a high burden of morbidity and mortality due to the limited availability of effective, safe and accessible diagnostic and therapeutic interventions (5,6,8), as well as the inability of local health systems to implement integrated surveillance and control programs in a sustainable and high-quality manner (2). Neglected tropical diseases persist as public health problems, resulting from the vulnerability conditions of populations that are, in fact, neglected (9).

In 2022, more than 1.5 billion people worldwide required some intervention related to the management of neglected tropical diseases (10). The elimination of this group of diseases is part of the global agenda of sustainable development goals, which requires integrated action across diseases and in an intersectoral manner (5,7,10).

Brazil has the highest burden of morbidity and mortality from neglected tropical diseases in Latin America (10). From 2016 to 2020, Chagas disease, schistosomiasis, Hansen disease, lymphatic filariasis, cutaneous leishmaniasis, visceral leishmaniasis, onchocerciasis, human rabies, trachoma and snakebites were responsible for 600 thousand cases, mainly in the North and Northeast regions (11). In the same period, a total of 30 million people were estimated to be at risk in the country – 14.0% of the Brazilian population (11).

The São Francisco River Integration Project may increase the risk and vulnerability to neglected tropical diseases in light of changes in the local epidemiological context. Monitoring areas influenced by this water infrastructure project is of great importance and must be carried out through the development of integrated strategic actions. The generation of evidence in this study may contribute to better decision-making through more consistent and effective health policies in the municipalities involved for the prevention and control of neglected tropical diseases. 

The main objective of this study was to estimate the prevalence of Chagas disease, Hansen disease and schistosomiasis among people living in rural productive villages of the São Francisco River Integration Project in Ceará and to verify the presence of triatomines, and *Trypanosoma cruzi* species and positivity status.

## Methods

### Study design

This is a descriptive cross-sectional study based on an integrated approach to three neglected tropical diseases using different methods and techniques: epidemiological survey for the diagnosis of human cases of schistosomiasis, Chagas disease and Hansen disease; and entomological survey with a focus on triatomines.

### Study area and population

The area covered by this study included the Goal 2N of the São Francisco River Integration Project, which is 39 kilometers long (12. The rural productive villages of Ipê (Jati – 14 houses), Descanso (Mauriti – 77 houses) and Vassouras (Brejo Santo – 154 houses) are located there, in Ceará State. The population of this study was made up of people living in areas directly affected by the project works and resettled in these villages.

In total, 810 people lived in the three rural productive villages (52 in Ipê, 275 in Descanso and 483 in Vassouras) during the study period. Of these, 180 household units (73.5%) received some social benefit, the most used means of family transport was the motorcycle (39.6%) and, considering the minimum wage of R$ 998.00 in 2019, the majority of families (68.6%) had an income between 1 and 2 minimum wages.

### Inclusion and exclusion criteria

The study included individuals of both sexes who agreed to participate in the research by signing the informed consent form or, when applicable, the informed assent form. Participants provided biological material (blood, feces and urine) in the quantity and quality necessary to carry out the research, were clinically evaluated and completed all data collection instruments for the research.

### Data collection

The data collection process for this research began with conversation circles in the communities of each rural productive village in July 2019. The research objectives and important aspects of each of the diseases that would be studied were presented during these conversation circles. In October 2019, visits to home units began, to apply records, characterization instruments and specific research related to diseases (collection of biological samples, entomological research, anamnesis and dermato-neurological examination). These activities in the villages continued until October 2020 and were attended by technicians and coordinators from the Ceará health department, from the health departments of the municipalities involved, professors and postgraduate students from the Federal University of Ceará with experience in the various activities of this study.

### Schistosomiasis surveys

Stool and urine samples were collected to identify cases of schistosomiasis. Two slides were prepared for each stool sample, using the Helm Test kit (Bio-Manguinhos, Fundação Oswaldo Cruz), based on the thick stool smear technique (13), following health surveillance recommendations (11).

Urine samples were analyzed according to the immunochromatographic standard regarding the presence of circulating cathodic antigen of *S. mansoni* (POC-CCA), using the Urine CCA kit (Schisto) ECO Test from ECO Diagnóstica, manufactured in Brazil. The manufacturer’s technical instructions were followed for these tests, taking as a reference for reading the intensity patterns of the immunochromatographic reaction using the G Score scale interpretation system (ranging from G1 to G10) (14). According to this classification, G1 are negative results (no appearance of the red band at the T line), G2 and G3 are considered trace (band at the T line with weak intensity), and, from G4 onwards, the results are considered positive with the band at the T line becoming increasingly more intense according to the score graduation. All tests require the appearance of C band (control band). For the purposes of results interpretation in this study, given the historical scenario of a low transmission area and the discussions in the literature on the interpretation of readings, the POC-CCA trace results were considered negative (15).

### Serological and entomological surveys for Chagas disease

For the identification of cases of Chagas disease, human blood samples were collected. The serological survey in rural productive villages was based on the enzyme-linked immunosorbent assay – ELISA (Chagatest Recombinant ELISA v. 3.0, Wiener Lab) with confirmation of reactive samples by indirect immunofluorescence – IFI (ImunoCON Chagas, WAMA Diagnostics). The diagnosis followed the recommendations of the 2015 Brazilian Consensus on Chagas Disease (16), of the Clinical Protocol and Therapeutic Guidelines for Chagas Disease (17), of the Brazilian Society of Cardiology (18) and surveillance by the Ministry of Health (11).

For the entomological survey, all households of the people included in the study were included. The research, the capture and analysis of triatomines, were carried out in partnership with the endemic disease coordinators of the three municipalities involved and with the Epidemiological Surveillance and Vector Control Cell, formerly the Vector Control Center of the Ceará State Health Department (11,19).

### Clinical-epidemiological survey for Hansen disease

The diagnostic evaluation was based on the anamnesis and dermato-neurological examination according to the protocols defined by the Ministry of Health for suspected cases and diagnostic confirmation (11,20). Suspected cases were referred for clinical evaluation by reference specialists, complemented by performing a skin smear for direct research (bacilloscopy) of *Mycobacterium leprae*.

### Data analysis

The results from the surveys were organized, tabulated and analyzed using KoBoToolbox – a free and open source set of field data collection tools that work online and offline (KoBoToolbox, Harvard Humanitarian Initiative, Cambridge, Massachusetts, United States). The descriptive analysis of the data was composed through the calculation of absolute and relative frequencies, in addition to central tendency measures. The prevalence estimates performed included the calculation of the 95% confidence interval (95%CI) using a binomial approximation of this proportion.

## Results

Regarding schistosomiasis, the Kato-Katz method was performed on 206 residents who provided stool samples. The mean estimated prevalence of schistosomiasis was 0.97% (2/206) – 95%CI, 0.12; 3.46%, being 3.57% (1/28) in the rural productive village of Ipê, 0.77% (1/130) in Vassouras and 0.00% (0/48) in Descanso. To perform the POC-CCA, 234 residents submitted urine samples, with a positivity rate of 11.53% (27/234). The highest positivity rate, not considering trait (G2 and G3) as positive, was 14.70% in Descanso (10/68), followed by 11.02% in Vassouras (14/127) and 7.69% in Ipê (3/39) (Table 1).

**Figure 1:**
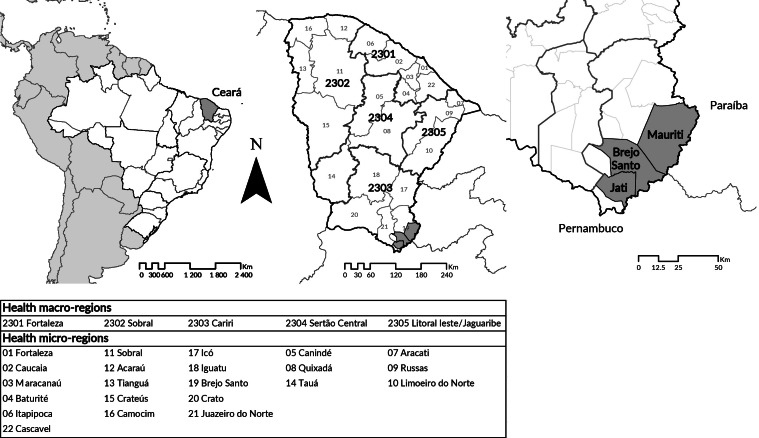
Study area, according to health macro and micro regions. Ceará, 2020

**Table 1:** Distribution of results of the Kato-Katz (n=206) and POC-CCA (n=234) methods for schistosomiasis by rural productive village in the study. Ceará, 2020

Rural productive village (municipality)	Kato-Katz				POC-CCA					
	Positive n (%)		Negative n (%)		Positive n (%)		Trace n (%)		Negative n (%)	
Ipê (Jati)	1	(3.57)	27	(96.43)	3	(7.70)	8	(20.51)	28	(71.79)
Descanso (Mauriti)	0	(0.00)	48	(100)	10	(14.71)	18	(26.47)	40	(58.82)
Vassouras (Brejo Santo)	1	(0.77)	129	(99.23)	14	(11.02)	13	(10.24)	100	(78.74)
Total	2	(0.97)	204	(99.03)	27	(11.54)	39	(16.67)	168	(71.79)

368 blood samples were collected to estimate the seroprevalence of Chagas disease among people living in rural productive villages (Table 2). The estimated overall prevalence was 0.27% (1/368) – 95%CI, 0.01; 1.50%.

**Table 2:** Distribution of results from enzyme immunoassay (ELISA) and indirect immunofluorescence (IFI) methods for Chagas disease by rural productive village in the study. Ceará, 2020 (n=368)

Rural productive village (municipality)	Samples collected	ELISA		IFI	
		Reactive n (%)	Non-reactive n (%)	Reactive n (%)	Non-reactive n (%)
Ipê (Jati)	42	0 (0.00)	42 (100)	0 (0.00)	42 (100)
Descanso (Mauriti)	136	4 (2.94)	132 (97.06)	1 (0.74)	135 (99.26)
Vassouras (Brejo Santo)	190	0 (0.00)	190 (100)	0 (0.00)	190 (100)
Total	368	4 (1.09)	364 (98.91)	1 (0.27)	367 (99.73)

In the analysis of triatomines, research was carried out following the regulations of the Ministry of Health (11), both inside and outside the homes of 245 households. In Ipê, 4 specimens of *Triatoma pseudomaculata* were found. In Descanso, 3 specimens were found: 2 specimens of *Triatoma pseudomaculata* and 1 of *Panstrongylus megistus*.In Vassouras, no specimen was found. After microscopic analysis of the feces of the collected triatomines, it was found that all were negative for T. cruzi. It is worth noting that all housing units were made of masonry with external wall plastering, tiled roofs and septic tanks (Table 3). 

**Table 3:** Dimensions of entomological surveillance in the households of residents of rural productive villages. Ceará, 2020 (n=245)

Variable	n (%)
**What materials were used to build the external walls of this home**?	
Masonry with external wall plastering	245 (100)
**What type of ceiling**?	
Tile	245 (100)
**How are feces and urine eliminated**?	
Septic tank	245 (100)
**What is the main source of water supply for this household**?	
General distribution network	155 (63.26)
Well or spring	90 (36.74)
**Are there chicken coops, corrals, piles of tiles or other outbuildings in the yard** (**peri-domiciliary space**)?	
Yes	158 (64.49)
No	87 (35.51)
**If so, which ones**?	
Chicken coop	153 (62.45)
Breeding pens	28 (11.43)
Pile of bricks or tiles	5 (2.04)
Pile of firewood or straw	4 (1.63)
Pile of wood	3 (1.22)
**Presence of domestic animals**?	
Yes	131 (53.47)
No	114 (46.53)
**If so, which ones**?	
Dog	114 (46.53)
Cat	43 (17.55)
Bird	4 (1.63)
**Presence of farm animals**?	
Yes	158 (64.49)
No	87 (35.51)
**If so, which ones**?	
Hen	150 (61.22)
Pork	17 (6.94)
Ox/cow	11 (4.49)
Sheep/Goat	10 (4.01)
Horse	9 (3.67)
Donkey/mule	8 (3.27)
Duck	4 (1.63)

300 clinical-epidemiological evaluations for Hansen disease were carried out (Ipê, 49; Descanso, 85; and Vassouras, 166), including dermato-neurological examination. Based on the identification of skin and peripheral nerves alterations during the examinations, 8 suspected cases were recognized in rural productive villages, 4 in Ipê, 1 in Descanso and 3 in Vassouras. All were referred for evaluation by reference specialists with collection of material by skin smear, with subsequent bacilloscopy. No cases have been confirmed.

## Discussion

This study demonstrated that territorial contexts of human occupation, post-2004, through the structuring of rural productive villages after the resettlement of populations affected by the São Francisco River Integration Project translates into the transposition of risk and vulnerability of rural residents to neglected tropical diseases, particularly in relation to schistosomiasis and Chagas disease. Located in a focal area for the transmission of these two diseases evaluated and Hansen disease, these villages also reflect contexts of restricted access to healthcare for these conditions, which may contribute to the persistence and amplification of transmission, even considering that occupation of these areas is recent.

Areas of the São Francisco River Integration Project located in semi-arid regions of the Brazilian Northeast, which could initially be considered to be at low risk for the occurrence of schistosomiasis, may in reality represent an increased potential risk of transmission due to the transposition of water. This occurs due to the possibility of presence and transposition of planorbids, which are hosts of S. mansoni, and of individuals susceptible to infection (21).

The importance of studying neglected tropical diseases such as schistosomiasis, Chagas disease and Hansen disease is unquestionable, considering the associated morbidity and mortality burden and the perpetuation of these diseases as a public health problem, often with territorial overlap (5,6,22). Such diseases are endemic in Brazil, especially in the Northeast of the country. This region has high levels of social vulnerability in most municipalities, with critical impacts on education and the subsistence economy, in addition to having low health care coverage and insufficient and critical basic sanitation conditions (5,7,23).

The impacts of the transposition of the São Francisco River create the challenge of defining strategic actions for monitoring and controlling these diseases in the areas directly affected by this work (2,24). There is a need to generate evidence that can effectively contribute to outlining actions, as well as defining the main risk areas to be worked on and raising awareness among managers to prioritize these populations (5).

Given this complex context and the impact caused by the construction of this large undertaking, it is essential to strengthen health education actions aimed at primary health care and health surveillance. The purpose is to encourage cooperation and strengthen the link between such actions, providing improved care for the population with more effective actions to combat neglected tropical diseases (2,5).

In Ceará State, the transposition of waters may lead to the introduction and colonization of the species *Biomphalaria glabrata* – the most effective intermediate host in the propagation of the etiological agent of schistosomiasis (2,25,26). This fact may contribute to the increased transmission of the disease, aggravated by the migration process between the cities that receive the construction sites for this project, with most of the migrants coming from endemic areas (27). The use of new diagnostic tools (POC-CCA) in this research demonstrated its potential and can provide faster responses for surveillance and health care actions in endemic territories (28), with a view to overcoming the high burden of morbidity and mortality (22).

In addition to the issues inherent to neglected tropical diseases, some factors ratified in this study may favor the occurrence of transmission of Chagas disease and triatomine species with significant potential for domiciliation and vectorial capacity. Historically, housing units prone to these diseases are built in a precarious manner: wattle and daub walls, mud walls and thatched roofs, wooden planks and unplastered masonry, making it easier for insects to adapt to these artificial habitats (16,19). In this study, even with 100.0% of the original houses in the project built to adequate standards (masonry with plaster, tile roof and septic tank), the presence of triatomines in these residences was evidenced and reported both inside and outside the home (19). These results suggest that masonry houses, which were previously considered “barriers” to the presence of insects inside the home, are subject to the presence of triatomines, requiring actions to prevent the access of vectors, especially at night, since artificial lighting can act as a source of attraction. The habit of raising birds and other animals in the peridomicile also favors the colonization of these insects, leading to the need for space management, as well as active surveillance in these areas (11,19).

Even though there were reports of confirmed cases of Hansen disease in the three municipalities studied, no cases were found in the rural productive villages. Since this is a chronic condition, strong participation of primary health care is required in monitoring its occurrence for the development of pertinent actions, particularly in endemic territorial contexts of greater social vulnerability (5,11). It is essential to strengthen operationalization in surveillance and control, aiming to establish actions to minimize potential risks in transmission.

Given the social determinants of this group of diseases, actions are needed that go beyond the perspective of the health sector (5,6). One of the most important factors to be worked on is improving the living conditions of communities that were deterritorialized to rural productive villages (2). Since the delivery of the village structures, the basic conditions necessary for families to sustain themselves from agriculture and animal husbandry, as they did previously, have not yet been guaranteed and provided.

Strengthening the institutional framework of the Brazilian Unified Health System becomes a preponderant factor for the execution of programs to control neglected tropical diseases, given the lack of prioritization of health surveillance and primary health care actions within municipalities after the process of decentralization of control actions (26).

A limitation of this study is the fact that most of the incursions into productive villages were carried out during the COVID-19 pandemic, which may have affected community mobilization. The estimated prevalences for neglected tropical diseases in the study were based on samples collected in 3 of 18 villages targeted by the project, which may limit the generalizability of the results to the broader population and may not adequately capture variation in prevalence in communities in other municipalities and states. Another limitation of this study refers to the factors inherent to the cross-sectional design, which are also limiting, given the impossibility of attesting causality and establishing temporal relationships.

In conclusion, Chagas disease and schistosomiasis persist as endemic in these areas. Even without the identification of cases of Hansen disease, the context of the region reinforces the maintenance of surveillance actions. There is a need for integration between surveillance and primary health care to control different neglected tropical diseases in new territories of human occupation. 

The integration between health surveillance and primary health care for different neglected tropical diseases in new occupation territories promoted by São Francisco River Integration Project in the municipalities analyzed is strategic for decision-making at all levels and spheres of management. The rural productive villages located in Jati, Mauriti and Brejo Santo are part of a larger endemic context within the municipal scope and are on alert for the possible occurrence of transmission of schistosomiasis, Chagas disease and Hansen disease.

Considering the contexts of endemicity and vulnerability for this group of diseases, it is essential to strengthen the inclusion of the topic in municipal and state public agendas for surveillance and control, including ongoing health education actions. The presence of infected individuals and triatomines indicates the need to enhance control actions and empower these communities. It is clear that training local health teams to tackle neglected tropical diseases is an essential factor, as is community participation in these actions. Strengthening the Brazilian Unified Health System through primary health care, integrated with health surveillance actions, is vital to achieving control.

## Data Availability

The database used in the research is available at: https://1drv.ms/x/c/e95b26732da697ea/EW5tAP3EwSdFnhePe_gRTBUBeuG1h55yImBSFngfSa704Q
