# Supplementary material for: Prevalence of neglected tropical diseases in rural productive villages of the São Francisco River Integration Project in Ceará: cross-sectional study, 2020
Source: Epidemiol Serv Saude. 2025 May 12;34:e20240393. doi: 10.1590/S2237-96222025v34e20240393.en (PMC12077539; doi:10.1590/S2237-96222025v34e20240393.en)
Supplement: Translation

# Translation: Prevalência de doenças tropicais negligenciadas em vilas produtivas rurais do Projeto de Integração do Rio São Francisco no Ceará: estudo transversal, 2020

**DOI:** 10.1590/S2237-96222025v34e20240393.pt

**Authors:** José Damião da Silva, Marta Cristhiany Cunha Pinheiro, Alberto Novaes Ramos, Bárbara Morgana da Silva, Anderson Fuentes Ferreira, Thainá Isabel Bessa de Andrade, Julieth Mesquita Lacerda, Letícia Pereira Araújo, Alanna Carla da Costa Belmino, Maria de Fátima Oliveira, Fernando Schemelzer de Moraes Bezerra

**Affiliations:** 1 Universidade Federal do Ceará, Faculdade de Medicina, Programa de Pós-Graduação em Saúde Pública, Fortaleza, CE, Brasil; 2 Universidade Federal do Ceará, Departamento de Análise Clínica e Toxicológica, Laboratório de Pesquisa em Parasitologia e Biologia de Moluscos, Fortaleza, CE, Brasil; 3 Universidade Federal do Ceará, Faculdade de Medicina, Departamento de Saúde Comunitária, Fortaleza, CE, Brasil; 4 Universidade Federal do Ceará, Departamento de Análises Clínicas e Toxicológicas, Laboratório de Pesquisa em Doença de Chagas, Fortaleza, CE, Brasil; 5 Universidade Federal do Ceará, Faculdade de Medicina, Programa de Pós-Graduação em Patologia, Fortaleza, CE, Brasil; 6 Universidade Federal do Ceará, Faculdade de Medicina, Programa de Pós-Graduação em Ciências Médicas, Fortaleza, CE, Brasil

**Keywords:** Doenças Negligenciadas, Vigilância em Saúde Pública, Atenção Primária à Saúde, Inquéritos Epidemiológicos, Estudos Transversais

## Abstract

**Objetivo:**

Estimar a prevalência de doença de Chagas, hanseníase e esquistossomose entre residentes de vilas produtivas rurais do Projeto de Integração do Rio São Francisco no Ceará e verificar a presença, espécie e positividade de triatomíneos para *Trypanosoma cruzi*.

**Métodos:**

Trata-se de estudo transversal descritivo nas vilas produtivas localizadas em Jati, Brejo Santo e Mauriti a partir de inquérito clínico-epidemiológico, sorológico e parasitológico para esquistossomose, doença de Chagas e hanseníase; e de inquérito triatomíneo. A análise descritiva foi composta por meio do cálculo de frequências absolutas e relativas com intervalos de confiança de 95%.

**Resultados:**

A prevalência de esquistossomose foi 0,97% (2/206) pelo método Kato-Katz e 11,54% (27/234) pelo método imunocromatográfico POC-CCA. Para doença de Chagas, foi 0,27% (1/368). Verificou-se 2,67% (8/300) de casos suspeitos de hanseníase por exame dermatoneurológico, nenhum confirmado posteriormente. Entre as 245 unidades domiciliares investigadas, em 4 (1,63%) foram identificados triatomíneos (6 *Triatoma pseudomaculata* e 1 *Panstrongylus megistus*), mas nenhum com presença de T. cruzi.

**Conclusões:**

Doença de Chagas e esquistossomose persistem como endemias nessas áreas do estudo. Mesmo sem a identificação de triatomíneos infectados por T. cruzi e de casos de hanseníase, o contexto da região reforça a necessidade de vigilância contínua. É fundamental implementar ações integradas de saúde pública para enfrentamento de diferentes doenças tropicais negligenciadas em novos territórios de ocupação humana. Contextos de endemicidade e vulnerabilidade tornam essencial o fortalecimento do tema nas agendas públicas municipais e estadual.

## Introdução

O processo de implantação de grandes empreendimentos hídricos invariavelmente acarreta significativas alterações ambientais, econômicas e socioculturais, que envolvem os mais dissonantes aspectos da dinâmica regional. Esse processo interfere no cotidiano das populações afetadas, envolvendo desterritorialização e modificação de seus modos de subsistência, sobretudo no tocante às condições de saúde e à qualidade de vida (1,2).

Em grandes obras hídricas, torna-se fundamental a percepção de que o represamento de águas ocasiona mudanças bruscas no ecossistema. Isso facilita o surgimento e a expansão de doenças, que podem, até o momento, não ser endêmicas nestas regiões (2,3). 

Nesse cenário, surgiu o Projeto de Integração do Rio São Francisco, empreendimento do governo federal que foi projetado para garantir segurança hídrica em 2025 para 12 milhões de habitantes de pequenas, médias e grandes cidades da região semiárida do Nordeste brasileiro. Para o alcance dessa meta, foram construídos dois canais: Eixo Norte, que leva água para os sertões de Pernambuco, do Ceará, da Paraíba e do Rio Grande do Norte, e Eixo Leste, que leva água para parte do sertão e região agreste de Pernambuco e da Paraíba (2,4). 

O projeto fomenta grandes preocupações no contexto da saúde pública, em especial por modificar as condições sociais e econômicas da vida das pessoas afetadas pelas obras, o que pode acarretar aumento na ocorrência de doenças associadas à pobreza, como as doenças tropicais negligenciadas (2,5,6). Estas compõem um grupo de 23 doenças causadas por vírus, bactérias, parasitas ou fungos, com padrão endêmico de ocorrência associado à pobreza (5,7).

Esse grupo de doenças se perpetua com elevada carga de morbimortalidade devido à limitada disponibilização de intervenções diagnósticas e terapêuticas eficazes, seguras e acessíveis (5,6,8), assim como pela incapacidade dos sistemas locais de saúde de implementar programas de vigilância e controle integrados, de forma sustentável e com qualidade (2). As doenças tropicais negligenciadas persistem como problemas de saúde pública, sendo resultado das condições de vulnerabilidade das populações que, de fato, são negligenciadas (9).

Em 2022, mais de 1,5 bilhão de pessoas no mundo precisaram de alguma intervenção relacionada ao manejo das doenças tropicais negligenciadas (10). A eliminação desse grupo de doenças insere-se na agenda global dos objetivos de desenvolvimento sustentável, o que demanda ação integrada entre as doenças e de maneira intersetorial (5,7,10).

O Brasil tem a maior carga de morbimortalidade por doenças tropicais negligenciadas na América Latina (10). De 2016 a 2020, doença de Chagas, esquistossomose, hanseníase, filariose linfática, leishmaniose tegumentar, leishmaniose visceral, oncocercose, raiva humana, tracoma e acidentes ofídicos foram responsáveis por 600 mil casos, principalmente nas regiões Norte e Nordeste (11). Nesse mesmo período, estimou-se no país o total de 30 milhões de pessoas sob risco – 14,0% da população brasileira (11).

O Projeto de Integração do Rio São Francisco pode ampliar o risco e a vulnerabilidade para doenças tropicais negligenciadas diante da mudança do contexto epidemiológico local. O monitoramento de áreas influenciadas por esse projeto de infraestrutura hídrico é de grande importância e deve ser realizado por meio do desenvolvimento de ações estratégicas integradas. A geração de evidências neste estudo poderá contribuir para a melhor tomada de decisões por meio de políticas de saúde mais consistentes e efetivas nos municípios envolvidos para prevenção e controle de doenças tropicais negligenciadas. 

O objetivo central deste estudo foi estimar a prevalência da doença de Chagas, hanseníase e esquistossomose entre pessoas residentes em vilas produtivas rurais do Projeto de Integração do Rio São Francisco no Ceará e verificar a presença de triatomíneos, espécie e positividade para *Trypanosoma cruzi*.

## Métodos

### Desenho do estudo

Trata-se de estudo transversal descritivo com base na abordagem integrada de três doenças tropicais negligenciadas por meio de diferentes métodos e técnicas: inquérito epidemiológico para diagnóstico de casos humanos de esquistossomose, doença de Chagas e hanseníase; e inquérito entomológico com foco em triatomíneos.

### Área e população do estudo

A área de atuação incluiu a Meta 2N do Projeto de Integração do Rio São Francisco com 39 quilômetros de extensão (12). Nela estão construídas as vilas produtivas rurais de Ipê (Jati – 14 casas), Descanso (Mauriti – 77 casas) e Vassouras (Brejo Santo – 154 casas), todas localizadas no Ceará. A população deste estudo foi composta por pessoas residentes das áreas diretamente afetadas pelas obras do projeto reassentados nessas vilas. 

Ao todo, 810 pessoas habitavam as três vilas produtivas rurais (52 em Ipê, 275 em Descanso e 483 em Vassouras) no período do estudo. Destes, em 180 unidades domiciliares (73,5%), havia o recebimento de algum benefício social, o meio de transporte familiar mais utilizado era a motocicleta (39,6%) e, considerando o salário mínimo de R$ 998,00 em 2019, a maioria das famílias (68,6%) possuía renda entre 1 e 2 salário mínimos.

### Critérios de inclusão e exclusão

Foram incluídos indivíduos de ambos os sexos, que concordaram em participar da pesquisa mediante a assinatura do registro do consentimento livre e esclarecido ou, quando aplicável, registro do assentimento livre e esclarecido. Os participantes forneceram material biológico (sangue, fezes e urina) em quantidade e qualidade necessárias para a realização da pesquisa, foram avaliados clinicamente e preencheram todos os instrumentos de coleta de dados para a pesquisa.

### Coleta de dados

O processo de coleta de dados desta pesquisa iniciou-se com a realização de rodas de conversa nas comunidades de cada vila produtiva rural em julho de 2019. Foram apresentados os objetivos da pesquisa e os aspectos importantes sobre cada uma das doenças que seriam trabalhadas. Em outubro de 2019, iniciaram-se as visitas às unidades domiciliares para aplicação dos registros, instrumentos de caracterização e pesquisas específicas relativas às doenças (coleta de amostras biológicas, pesquisa entomológica, anamnese e exame dermatoneurológico). Essas atividades nas vilas se estenderam até outubro de 2020 e contaram com a participação de técnicos e coordenadores da secretaria da saúde do Ceará, das secretarias de saúde dos municipais envolvidos, docente e discentes de pós-graduação da Universidade Federal do Ceará com experiência nas diversas atividades deste estudo. 

### Inquéritos para esquistossomose

Para a identificação de casos de esquistossomose, foram coletadas amostras de fezes e urina. Confeccionaram-se duas lâminas para cada amostra de fezes, utilizando-se para análise o kit Helm Test (Bio-Manguinhos, Fundação Oswaldo Cruz), com base na técnica de esfregaço espesso de fezes (13), seguindo as recomendações de vigilância em saúde (11).

As amostras de urina foram analisadas segundo padrão imunocromatográfico quanto à presença de antígeno catódico circulante de *S. mansoni* (POC-CCA), utilizando-se o kit Urine CCA (Schisto) ECO Teste da ECO Diagnóstica, fabricado no Brasil. As instruções técnicas do fabricante foram seguidas para a realização dos testes, tomando-se como referência para a leitura os padrões de intensidade da reação imunocromatográfica utilizando o sistema de interpretação em escala de Score G (variando de G1 a G10) (14). Segundo essa classificação, G1 são os resultados negativos (sem aparecimento da banda T), G2 e G3 são considerados traço (banda T com fraca intensidade), e, a partir de G4, os resultados são considerados positivos com a banda T ficando cada mais intensa de acordo com a graduação do escore. Todos os testes possuem a obrigatoriedade do aparecimento da banda C (banda controle). Para fins de interpretação dos resultados neste estudo, considerando o cenário histórico de área de baixa transmissão e as discussões na literatura sobre a interpretação de leituras, os resultados POC-CCA traço foram considerados negativos (15).

### Inquéritos sorológico e entomológico para doença de Chagas

Para a identificação dos casos de doença de Chagas, foram coletadas amostras de sangue humano. O inquérito sorológico nas vilas produtivas rurais baseou-se no ensaio imunoenzimático – ELISA (Chagatest ELISA recombinante v. 3.0, Wiener Lab) com confirmação das amostras reagentes por meio da imunofluorescência indireta – IFI (ImunoCON Chagas, WAMA Diagnóstica). O diagnóstico seguiu as recomendações do Consenso Brasileiro em Doenças de Chagas de 2015 (16), do Protocolo Clínico e Diretrizes Terapêuticas em Doença de Chagas (17), da Sociedade Brasileira de Cardiologia (18) e de vigilância do Ministério da Saúde (11).

Para o inquérito entomológico, foram incluídas todas as unidades domiciliares das pessoas incluídas no estudo. A pesquisa, a captura e a análise dos triatomíneos foram realizadas em parceria com as coordenações de endemias dos três municípios envolvidos e com a Célula de Vigilância Epidemiológica e Controle de Vetores, antigo Núcleo de Controle de Vetores da Secretaria de Saúde do Estado do Ceará (11,19).

### Inquérito clínico-epidemiológico para hanseníase

A avaliação diagnóstica baseou-se na anamnese e no exame dermatoneurológico de acordo com os protocolos definidos pelo Ministério da Saúde para suspeição e confirmação diagnóstica (11,20). Casos suspeitos foram encaminhados para avaliação clínica por especialistas de referência, complementada pela realização de esfregaço dérmico para a pesquisa direta (baciloscopia) de *Mycobacterium leprae*.

### Análise dos dados

Os resultados obtidos dos inquéritos foram organizados, tabulados e analisados por meio do KoBoToolbox – conjunto de ferramentas para coleta de dados de campo, gratuito e de código aberto com funcionamento on-line e off-line (KoBoToolbox, Harvard Humanitarian Initiative, Cambridge, Massachusetts, Estados Unidos). A análise descritiva dos dados foi composta por meio do cálculo de frequências absolutas e relativas, além de medidas de tendência central. As estimativas de prevalência realizadas incluíram o cálculo do intervalo de confiança de 95% (IC95%) por meio de aproximação binomial dessa proporção.

## Resultados

Em relação à esquistossomose, o método Kato-Katz foi realizado em 206 moradores que disponibilizaram amostras de fezes. A estimativa média de prevalência da esquistossomose foi 0,97% (2/206) – IC95% 0,12; 3,46%, sendo 3,57% (1/28) na vila produtiva rural Ipê, 0,77% (1/130) em Vassouras e 0,00% (0/48) em Descanso. Para realização do POC-CCA, 234 moradores entregaram amostra de urina, com proporção de positividade de 11,53% (27/234). A maior taxa de positividade, não considerando traço (G2 e G3) como positivo, foi 14,70% (10/68) em Descanso, seguida por 11,02% (14/127) em Vassouras e 7,69% (3/39) em Ipê (Tabela 1).

**Figura 1:** Área do estudo, segundo macro e microrregiões de saúde. Ceará, 2020

**Tabela 1:** Distribuição dos resultados dos métodos Kato-Katz (n=206) e POC-CCA (n=234) para esquistossomose por vila produtiva rural do estudo. Ceará, 2020

Vila produtiva rural (município)	Kato-Katz				POC-CCA					
	Positivo n (%)		Negativo n (%)		Positivo n (%)		Traço n (%)		Negativo n (%)	
Ipê (Jati)	1	(3,57)	27	(96,43)	3	(7,70)	8	(20,51)	28	(71,79)
Descanso (Mauriti)	0	(0,00)	48	(100)	10	(14,71)	18	(26,47)	40	(58,82)
Vassouras (Brejo Santo)	1	(0,77)	129	(99,23)	14	(11,02)	13	(10,24)	100	(78,74)
Total	2	(0,97)	204	(99,03)	27	(11,54)	39	(16,67)	168	(71,79)

Foram coletadas 368 amostras de sangue para estimar a soroprevalência da doença de Chagas entre as pessoas residentes nas vilas produtivas rurais (Tabela 2). A prevalência geral estimada foi 0,27% (1/368) – IC95% 0,01; 1,50%.

**Tabela 2:** Distribuição dos resultados dos métodos ensaio imunoenzimático (ELISA) e imunofluorescência indireta (IFI) para doença de Chagas por vila produtiva rural do estudo. Ceará, 2020 (n=368)

Vila produtiva rural (município)	Amostras coletadas	ELISA		IFI	
		Reativa n (%)	Não reativa n (%)	Reativa n (%)	Não reativa n (%)
Ipê (Jati)	42	0 (0,00)	42 (100)	0 (0,00)	42 (100)
Descanso (Mauriti)	136	4 (2,94)	132 (97,06)	1 (0,74)	135 (99,26)
Vassouras (Brejo Santo)	190	0 (0,00)	190 (100)	0 (0,00)	190 (100)
Total	368	4 (1,09)	364 (98,91)	1 (0,27)	367 (99,73)

Na análise dos triatomíneos, foram realizadas pesquisas seguindo as normativas do Ministério da Saúde (11), tanto no intra quanto no peridomicílio de 245 unidades domiciliares. Em Ipê, foram encontrados 4 exemplares de *Triatoma pseudomaculata*. Em Descanso, foram encontrados 3 espécimes: 2 exemplares de T. pseudomaculata e 1 de *Panstrongylus megistus*. Em Vassouras, não foi encontrado nenhum exemplar. Após análises microscópicas das fezes dos triatomíneos coletados, verificou-se que todos estavam negativos para T. cruzi. Vale destacar que todas as unidades domiciliares eram de alvenaria com revestimento (reboco), com cobertura de telha e com fossa séptica (Tabela 3). 

**Tabela 3:** Dimensões de vigilância entomológica nas unidades domiciliares dos moradores das vilas produtivas rurais. Ceará, 2020 (n=245)

Variável	n (%)
**Quais os materiais na construção das paredes externas deste domicílio**?	
Alvenaria com revestimento (reboco)	245 (100)
**Qual o tipo de teto**?	
Telha	245 (100)
**De que forma são eliminados as fezes e urina**?	
Fossa séptica	245 (100)
**Qual é a principal forma de abastecimento de água deste domicílio**?	
Rede geral de distribuição	155 (63,26)
Poço ou nascente	90 (36,74)
**Existem galinheiros, currais, amontoados de telhas ou outros anexos no quintal** (**espaço peridomiciliar**)?	
Sim	158 (64,49)
Não	87 (35,51)
**Caso sim**, quais?	
Galinheiro	153 (62,45)
Currais de criação	28 (11,43)
Amontoado de tijolos ou telhas	5 (2,04)
Amontoado de lenha ou palhas	4 (1,63)
Amontoado de madeiras	3 (1,22)
**Presença de animais domésticos**?	
Sim	131 (53,47)
Não	114 (46,53)
**Caso sim**, quais?	
Cão	114 (46,53)
Gato	43 (17,55)
Pássaro	4 (1,63)
**Presença de animais de criação**?	
Sim	158 (64,49)
Não	87 (35,51)
**Caso sim**, quais?	
Galinha	150 (61,22)
Porco	17 (6,94)
Boi/vaca	11 (4,49)
Carneiro/bode	10 (4,01)
Cavalo	9 (3,67)
Burro/jumento/mula	8 (3,27)
Pato	4 (1,63)

Foram realizadas 300 avaliações clínico-epidemiológicas para hanseníase (Ipê – 49; Descanso – 85; e Vassouras – 166), incluindo o exame dermatoneurológico. A partir da identificação de alterações na pele e em nervos periféricos durante os exames, foram reconhecidos 8 casos suspeitos nas vilas produtivas rurais, sendo 4 em Ipê, 1 em Descanso e 3 em Vassouras. Todos foram encaminhados para avaliação com especialistas de referência com coleta de material por esfregaço dérmico, com posterior realização de baciloscopia. Nenhum caso foi confirmado.

## Discussão

Este estudo demonstrou que contextos territoriais de ocupação humana, pós-2004, por meio da estruturação das vilas produtivas rurais a partir do reassentamento de populações atingidas pelo Projeto de Integração do Rio São Francisco traduzem a transposição de risco e vulnerabilidade de moradores rurais para doenças tropicais negligenciadas, particularmente em relação à esquistossomose e à doença de Chagas. Localizadas em área focal para transmissão dessas duas doenças avaliadas e da hanseníase, essas vilas também refletem contextos de restrição de acesso à saúde para essas condições, o que pode contribuir para a persistência e a amplificação da transmissão, mesmo considerando-se ser novas áreas de ocupação. 

Áreas do Projeto de Integração do Rio São Francisco situadas em regiões semiáridas do Nordeste brasileiro, que inicialmente poderiam ser consideradas de baixo risco para a ocorrência de esquistossomose, na realidade podem representar um risco potencial acrescido de transmissão pela transposição de águas. Isso ocorre devido à possiblidade de presença e transposição também de planorbídeos, que são hospedeiros de S. mansoni, e de indivíduos suscetíveis à infecção (21).

É inquestionável a importância do estudo de doenças tropicais negligenciadas como esquistossomose, doença de Chagas e hanseníase, considerando-se a carga de morbimortalidade associada e a perpetuação dessas doenças como problema de saúde pública, muitas vezes em superposição territorial (5,6,22). Tais doenças são endêmicas no Brasil, em especial no Nordeste do país. Essa região possui altos índices de vulnerabilidade social na maioria dos municípios, com impactos críticos na educação e na economia de subsistência, além de possuir baixas coberturas de atenção à saúde e condições críticas e insuficientes de saneamento básico (5,7,23).

Os impactos da transposição do rio São Francisco geram o desafio de definir ações estratégicas de vigilância e controle dessas doenças nas áreas diretamente afetadas por essa obra (2,24). Faz-se necessária a geração de evidências que possam contribuir efetivamente para delinear ações, como também definir as principais áreas de risco a serem trabalhadas e sensibilizar gestores para priorização dessas populações (5).

Diante desse complexo contexto e do impacto causado pela construção desse grande empreendimento, é fundamental o fortalecimento das ações de educação em saúde direcionadas à atenção primária à saúde e vigilância em saúde. A finalidade é de estimular a cooperação e fortalecer o vínculo entre tais ações, proporcionando melhoria da atenção prestada à população com ações mais efetivas para o enfrentamento das doenças tropicais negligenciadas (2,5).

A transposição das águas pode ocasionar a introdução e colonização, no Ceará, da espécie *Biomphalaria glabrata* – hospedeiro intermediário mais efetivo na propagação do agente etiológico da esquistossomose (2,25,26). Esse fato pode contribuir para o aumento na transmissão da doença, agravado pelo processo migratório entre as cidades que recebem os canteiros de obras desse projeto, vindos em sua maioria de áreas endêmicas (27). O uso de novas ferramentas para diagnóstico (POC-CCA) nesta pesquisa demonstrou seu potencial e pode oportunizar respostas mais rápidas para as ações de vigilância e atenção à saúde nos territórios endêmicos (28), com vistas a superar as elevadas cargas de morbimortalidade (22).

Para além das questões inerentes às doenças tropicais negligenciadas, alguns fatores ratificados neste estudo podem favorecer a ocorrência de transmissão da doença de Chagas e de espécies de triatomíneos com importante potencial de domiciliação e capacidade vetorial. Historicamente, as unidades domiciliares propícias são construídas de forma precária: paredes de pau a pique, barreadas e com telhado de sapê, tábuas de madeira e alvenaria sem reboco, facilitando a adaptação dos insetos a esses habitats artificiais (16,19). Neste estudo, mesmo com 100,0% das casas originais do projeto construídas em padrões adequados (alvenaria com reboco, cobertura de telha e fossa séptica), foi evidenciada e relatada a presença de triatomíneos nessas residências tanto no intra quanto no peridomicílio (19). Esses resultados sugerem que as moradias de alvenaria, que antes eram consideradas “barreiras” para o encontro de insetos no intradomicílio, estão sujeitas à presença dos triatomíneos, necessitando de ações para dificultar o acesso dos vetores, principalmente à noite, já que a iluminação artificial pode atuar como fonte de atração. O hábito de criar aves e outros animais no peridomicílio também favorece a colonização desses insetos, levando à necessidade de gestão desse espaço, assim como vigilância ativa nessas áreas (11,19).

Mesmo havendo notificações de casos confirmados de hanseníase nos três municípios estudados, nenhum caso foi encontrado nas vilas produtivas rurais. Como condição crônica, demanda-se a forte participação da atenção primária à saúde no monitoramento de sua ocorrência para o desenvolvimento de ações pertinentes, particularmente em contextos territoriais endêmicos de maior vulnerabilidade social (5,11). Torna-se imprescindível o fortalecimento da operacionalização na vigilância e no controle, visando ao estabelecimento de ações no sentido de minimizar potenciais riscos na transmissão.

Diante dos determinantes sociais desse grupo de doenças, são necessárias ações que ultrapassem a perspectiva do setor saúde (5,6). Um dos fatores mais importantes a ser trabalhado é a melhoria nas condições de vida das comunidades que foram desterritorializadas para as vilas produtivas rurais (2). Desde a entrega das estruturas das vilas, ainda não foram garantidas e fornecidas as condições básicas necessárias para as famílias se manterem da agricultura e da criação de animais, como faziam anteriormente.

O fortalecimento do arcabouço institucional do Sistema Único de Saúde torna-se fator preponderante para a execução de programas de controle de doenças tropicais negligenciadas, diante da não priorização das ações de vigilância em saúde e atenção primária à saúde no âmbito dos municípios após o processo de descentralização das ações de controle (26).

Entende-se como limitação deste estudo o fato de as incursões às vilas produtivas terem sido realizadas, em sua maioria, no período da pandemia da covid-19, o que pode ter afetado a mobilização comunitária. As prevalências estimadas para as doenças tropicais negligenciadas do estudo foram baseadas em amostras coletadas em 3 de 18 vilas previstas pelo projeto, o que pode limitar a generalização dos resultados para a população mais ampla e pode não capturar adequadamente a variação da prevalência nas comunidades de outros municípios e estados. Outra limitação deste estudo refere-se aos fatores inerentes ao delineamento transversal que também são limitantes, dada a impossibilidade de atestar causalidade e estabelecer relações temporais.

Conclui-se que a doença de Chagas e a esquistossomose persistem como endemias nessas áreas. Mesmo sem a identificação de casos de hanseníase, o contexto da região reforça a manutenção de ações de vigilância. Ressalta-se a necessidade de integração entre vigilância e atenção primária à saúde para controle de diferentes doenças tropicais negligenciadas em novos territórios de ocupação humana. 

A integração entre vigilância em saúde e atenção primária à saúde para diferentes doenças tropicais negligenciadas em novos territórios de ocupação promovidos pelo Projeto de Integração do Rio São Francisco nos municípios analisados é estratégica para tomada de decisões em todos os níveis e as esferas de gestão. As vilas produtivas rurais localizadas em Jati, Mauriti e Brejo Santo inserem-se em contexto endêmico maior no âmbito municipal e de alerta para a possível ocorrência de transmissão de esquistossomose, doença de Chagas e hanseníase.

Considerando-se os contextos de endemicidade e vulnerabilidade para esse grupo de doenças, torna-se fundamental fortalecer a inclusão do tema nas agendas públicas municipais e estadual para vigilância e controle, incluindo ações de educação permanente em saúde. A presença de indivíduos infectados e de triatomíneos indica a necessidade de potencializar as ações de controle e o empoderamento dessas comunidades. Fica claro que a capacitação das equipes de saúde locais para enfrentamento das doenças tropicais negligenciadas é fator imprescindível, assim como a participação da comunidade nessas ações. O fortalecimento do Sistema Único de Saúde a partir da atenção primária à saúde, de modo integrado às ações da vigilância em saúde, é vital para o alcance do controle.

## Data Availability

O banco de dados utilizado na pesquisa está disponível em: https://1drv.ms/x/c/e95b26732da697ea/EW5tAP3EwSdFnhePe_gRTBUBeuG1h55yImBSFngfSa704Q
